# Altered directional functional connectivity underlies post-stroke cognitive recovery

**DOI:** 10.1093/braincomms/fcad149

**Published:** 2023-05-06

**Authors:** Behrad Soleimani, Isabella Dallasta, Proloy Das, Joshua P Kulasingham, Sophia Girgenti, Jonathan Z Simon, Behtash Babadi, Elisabeth B Marsh

**Affiliations:** Department of Electrical and Computer Engineering, University of Maryland, College Park, MD 20742, USA; Institute for Systems Research, University of Maryland, College Park, MD 20740, USA; Department of Neurology, the Johns Hopkins School of Medicine, Baltimore, MD 21287, USA; Department of Anesthesia, Critical Care and Pain Medicine, Massachusetts General Hospital, Boston, MA 02114, USA; Department of Electrical Engineering, Linköping University, SE-581 83 Linköping, Sweden; Department of Neurology, the Johns Hopkins School of Medicine, Baltimore, MD 21287, USA; Department of Electrical and Computer Engineering, University of Maryland, College Park, MD 20742, USA; Institute for Systems Research, University of Maryland, College Park, MD 20740, USA; Department of Biology, University of Maryland, College Park, MD 20742, USA; Department of Electrical and Computer Engineering, University of Maryland, College Park, MD 20742, USA; Institute for Systems Research, University of Maryland, College Park, MD 20740, USA; Department of Neurology, the Johns Hopkins School of Medicine, Baltimore, MD 21287, USA

**Keywords:** stroke recovery, functional connectivity, MEG, granger causality, cognition

## Abstract

Cortical ischaemic strokes result in cognitive deficits depending on the area of the affected brain. However, we have demonstrated that difficulties with attention and processing speed can occur even with small subcortical infarcts. Symptoms appear independent of lesion location, suggesting they arise from generalized disruption of cognitive networks. Longitudinal studies evaluating directional measures of functional connectivity in this population are lacking. We evaluated six patients with minor stroke exhibiting cognitive impairment 6–8 weeks post-infarct and four age-similar controls. Resting-state magnetoencephalography data were collected. Clinical and imaging evaluations of both groups were repeated 6- and 12 months later. Network Localized Granger Causality was used to determine differences in directional connectivity between groups and across visits, which were correlated with clinical performance. Directional connectivity patterns remained stable across visits for controls. After the stroke, inter-hemispheric connectivity between the frontoparietal cortex and the non-frontoparietal cortex significantly increased between visits 1 and 2, corresponding to uniform improvement in reaction times and cognitive scores. Initially, the majority of functional links originated from non-frontal areas contralateral to the lesion, connecting to ipsilesional brain regions. By visit 2, inter-hemispheric connections, directed from the ipsilesional to the contralesional cortex significantly increased. At visit 3, patients demonstrating continued favourable cognitive recovery showed less reliance on these inter-hemispheric connections. These changes were not observed in those without continued improvement. Our findings provide supporting evidence that the neural basis of early post-stroke cognitive dysfunction occurs at the network level, and continued recovery correlates with the evolution of inter-hemispheric connectivity.

## Teaser

Patients with minor stroke can exhibit cognitive difficulties due to disrupted cognitive networks. Marsh, et al. longitudinally evaluated directional functional connectivity in 6 patients and 4 controls. During early recovery, there were increased inter-hemispheric connections directed from the ipsilesional to the contralesional cortex that further evolved only in those with better long-term clinical outcomes.

## Graphical Abstract

**Graphical abstract:**
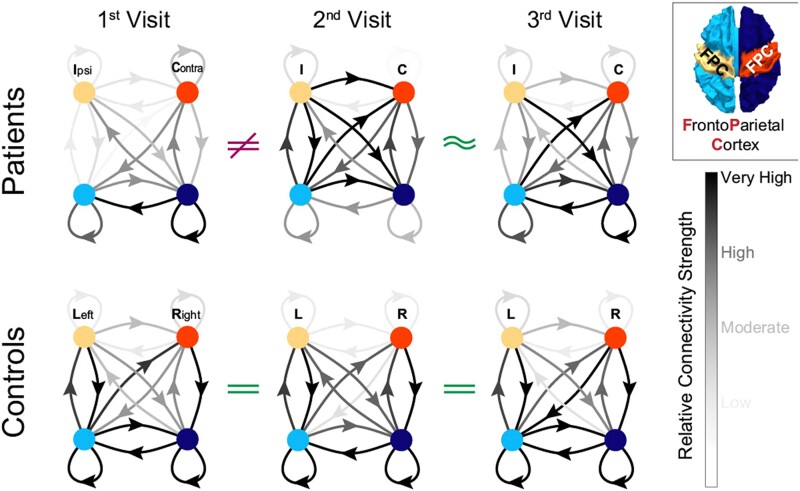

## Introduction

Stroke is one of the leading causes of long-term disability world-wide.^1^ Significant advances in both acute stroke care and rehabilitation have resulted in improved functional outcomes of motor and language deficits.^2,3^ Due to intravenous thrombolysis and mechanical thrombectomy, patients presenting with large hemispheric areas of ischaemia are being treated and discharged with significantly smaller infarcts.^4–8^ This has changed the landscape of stroke recovery, altering the most common presentations of post-stroke deficits. Unfortunately, despite sparing large cortical regions, patients with smaller, often subcortical, ‘minor strokes’ can nevertheless demonstrate significant difficulties with attention, multi-tasking, processing speed and other executive functions.^9,10^

Vascular cognitive impairment is well described in the literature.^11–15^ However, it is typically characterized by an accumulation of infarcts manifesting as a stepwise decline, or stroke involving a large cortical area traditionally felt to be responsible for various cognitive functions such as language or attention. In contrast, recent studies have demonstrated that even single small infarcts, independent of lesion location, can lead to impairment, resulting in a dysexecutive syndrome that demonstrates variable recovery.^9,16,17^ This constellation of cognitive symptoms occurs reliably within the minor stroke population^18^ and can be disabling, preventing patients from returning to work and living normal lives.^19^ Our previous neuroelectrophysiological work using magnetoencephalography (MEG) has shown temporal dispersion of evoked responses during cognitive tasks independent of infarct size or location in this group, suggesting that minor strokes disrupt cognitive function by ‘lesioning the network’.^17,20^ To date, formal connectivity studies to explain the underlying aetiology of post-stroke cognitive dysfunction after a minor stroke, specifically those evaluating directional connectivity between key areas of the cortex, are lacking.

Fortunately, despite early cognitive difficulties, many patients with minor stroke recover well. By six months after infarct, the majority have significantly improved clinically.^19^ Interestingly, however, findings of temporal dispersion and alterations in beta-band activity are still seen on MEG at this time point despite functional improvement.^17,20^ The mechanism by which many recover remains poorly elucidated. In addition, the longer-term trajectory of these patients is variable. Some individuals continue to improve while others revert to worsened cognitive performance.^19^ The underlying neurophysiology warrants further investigation.

This small proof-of-concept study is the logical next step to evaluate the role of functional connectivity in the longitudinal cognitive recovery of patients following minor stroke. To explore the hypothesis that acute cognitive impairment following small infarcts is due to network dysfunction and that specific patterns of network evolution over time are linked to favourable long-term recovery, we formally analyse directional functional connectivity, the influence that one area of the brain exerts on another, using resting-state MEG data from patients with minor stroke collected longitudinally at three visits, each approximately six months apart. Directional functional connectivity can best be described as the relationship between the activity of neurons in group A following the earlier activation of group B and can be measured using Network Localized Granger Causality (NLGC).^21^ Functional MRI (fMRI) studies evaluating predominantly larger cortical strokes and focused on language and motor impairment have demonstrated subsequent recovery with improved functional connectivity;^22,23^ however, few have explored how different directional connectivity relationships may influence outcome. Since cognitive processes generally occur on a rapid scale, for this study we instead use MEG to evaluate connectivity, which allows for the evaluation of neural activity, and hence directional connectivity, on a millisecond scale. The utility of MEG to study post-stroke cognition has been demonstrated in a recent prior study focused on larger, hemispheric lesions.^24^ To avoid the influence of severe hemiparesis or aphasia on clinical assessment, however, only patients with minor strokes were included in this study. While the definition of minor stroke varies throughout the literature based on stroke severity versus vascular involvement,^8,25,26^ our inclusion criteria focus on small, predominantly subcortical, ischaemic infarcts, allowing for evaluation of generalized disruption of cognitive networks without the confounding effect of direct cortical involvement.

## Materials and methods

### Subjects and cognitive assessment

**Figure 1:**
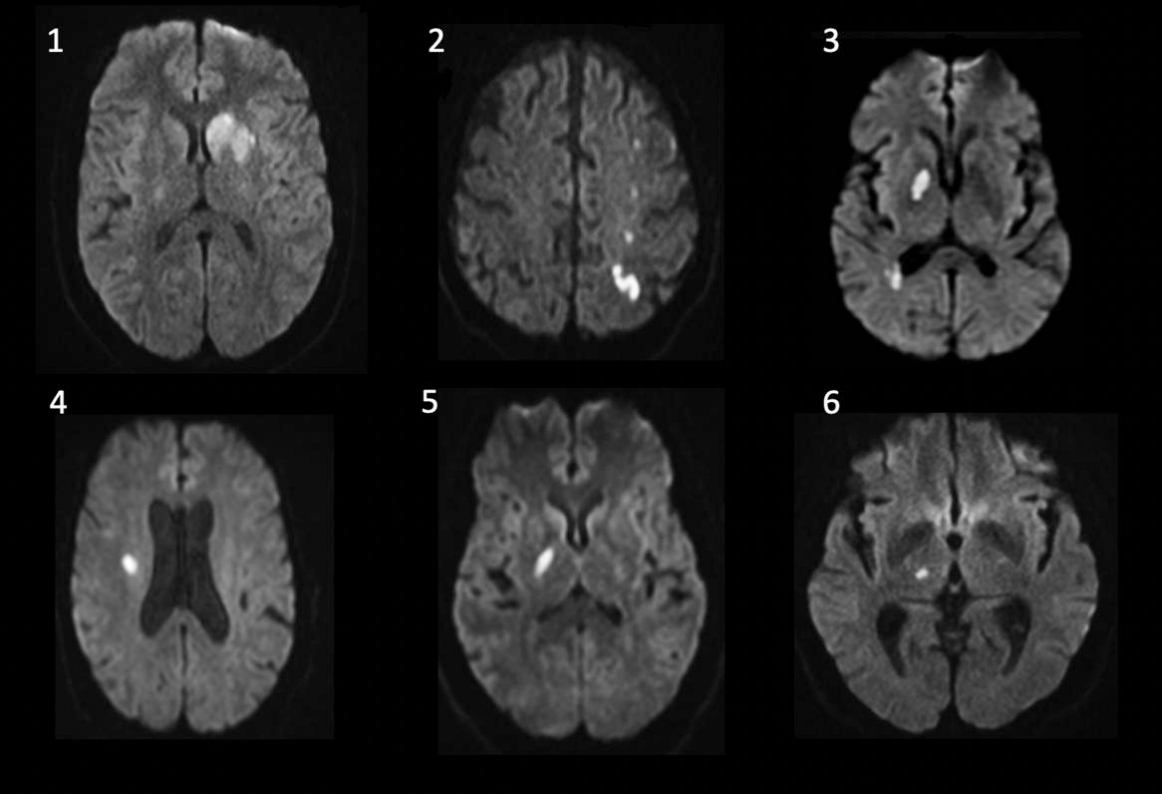
**MRI scans.** Representative diffusion weighed MRI scans of patients 1–6 (*N* = 6) depicting their small, predominantly subcortical infarcts.

This study was approved by the Johns Hopkins University institutional review board and all participants provided written informed consent. Resting-state MEG data were collected from six patients returning for follow-up 6–8 weeks after hospitalization (visit 1) for their first ever minor acute ischaemic stroke, and four controls (age-matched within five years) without a history of prior stroke or neurologic disease. Minor stroke was defined as an admission National Institutes of Health Stroke Scale (NIHSS) score^27^ of ten or less (higher than some definitions in order to allow for deep small vessel lacunar infarcts resulting in initial dysarthia, weakness, and sensory loss), with no large vessel territory involvement (e.g. M1 or M2 occlusion), significant hemiparesis, aphasia or hemispatial neglect. Of note, for those recruited, NIHSS scores were all significantly below the inclusion threshold (all <4) at the first follow-up visit. Infarct location and stroke volume were determined using diffusion-weighted MRI (see Fig. 1). In addition, patients were required to have a good pre-stroke baseline [modified Rankin score (mRS)^28^ of two or less], and no history of previously documented dementia or current untreated psychiatric illness. Non-native English speakers were also excluded, along with those with prior clinical stroke, and uncorrected hearing or visual loss. Subjects included in this study are the subset of a population originally reported in Marsh *et al*.^17^ and Kulasingham *et al*.^20^ who returned for both their 6- and 12 month follow-up visits (visits 2 and 3), allowing for formal functional connectivity analyses and longitudinal investigation corresponding to clinical change. The remaining participants were unable to be seen due to the COVID pandemic, and, therefore, were not included in this analysis.

Clinical performance, along with additional demographic and stroke characteristics including Montreal Neurological Institute coordinates of the middle of the largest area of infarct, are detailed in Table 1. All patients underwent a comprehensive neurological examination and demonstrated no evidence of difficulty with reading, writing, naming, or comprehending written or spoken stimuli, and only mild weakness, if any, at the time of the first follow-up. Although these patients exhibited no significant hemiparesis, at visit 1 they displayed mild bilateral motor deficits in the form of reduced dexterity and slowed reaction times along with mild cognitive impairment on the Montreal Cognitive Assessment (MoCA)^29^ that improved by the second visit. A neuropsychological battery was performed focusing on visual memory [Hopkins verbal learning test (HVLT)-revised: immediate and delayed recall], attention and executive function [Delis-Kaplan Executive Function System (D-KEFS): verbal fluency and trails making tests], and processing speed [symbol digit modalities test (SDMT)]. Additional variables were collected at each visit including the NIHSS score evaluating stroke severity, and the mRS and Barthel Index evaluating functional performance and activities of daily living. Z scores were generated using normative data for each task. A patient was considered to be impaired for that test/domain if they scored >1.5 standard deviations below the mean. Change in score over time was also evaluated, with particular attention paid to the interval between visits 2 and 3 (Table 1), as this time period of recovery has been shown to be the most variable, with not all patients continuing to show improvement.^19^ Patients were characterized as having a ‘favourable’ long-term recovery profile at visit 3 if they showed improvement in greater than half of the tests/domains compared to visit 2.

**Table 1:** Patient characteristics

Stroke participant	Stroke characteristics			Follow-up	Severity	Function			Verbal Memory	Executive function		Processing speed		Long-term outcome
	Hemisphere (Montreal Neurological Institute coordinates)	Volume (cc)	Acute NIHSS	Visit	NIHSS	mRS	Barthel Index	MoCA	HVLT	Verbal fluency	Trail making	Ipsilesional pegboard	SDMT	Favourable recovery profile
**1**	Left	9.8	4	1	0	1	100	24	−2.000	−0.867	−1.760	−1.480	−1.410	
*43 yo white woman*	(*x*: –23.72, *y*: 5.73, *z*: 3.96)			2	0	1	100	26	0.600	0.000	−0.120	−2.830	−0.790	
				3	0	1	100	27	−0.600	0.333	0.200	−1.030	−1.210	Yes
**2**	Left	1.7	0	1	0	1	100	26	−2.550	2.100	0.200	−3.250	0.770	
*79 yo white man*	(*x*: −26.91, *y*: −50.93, *z*: 53.14)			2	0	1	100	27	−1.350	2.100	0.660	−4.140	0.530	
				3	0	0	100	28	−0.350	3.000	0.540	−3.620	0.300	Yes
**3**	Right	9.4	3	1	3	1	100	21	−2.300	−0.867	−2.140	−5.030	−0.560	
*70 yo white man*	(*x*: 17.51, *y*: 1.19, *z*: 7.02)			2	0	1	100	20	−2.450	−1.067	−1.200	−8.360	−1.340	
				3	0	1	100	20	−2.200	−1.200	−1.080	−3.260	−1.730	No
**4**	Right	0.3	2	1	2	1	100	24	−2.600	−1.633	−2.400	−15.680	−1.800	
*56 yo black woman*	(*x*: 31.07, *y*: −4.32, *z*: 24.46)			2	0	0	100	29	−1.900	−0.667	−1.400	−0.380	−1.030	
				3	0	0	100	27						No
**5**	Right	0.8	3	1	0	1	100	26	−1.900	−0.233	−0.960	−2.090	−1.100	
*37 yo black man*	(*x*: 16.77, *y*: −7.56, *z*: 10.02)			2	0	0	100	27	−2.250	−0.330	0.000	−0.780	−1.310	
				3	0	0	100	29	−0.200	0.333	0.220	0.850	−1.000	Yes
**6**	Right	0.3	1	1	1	0	100	28	−1.200	−0.333	−1.080	−7.810	−0.720	
*66 yo white woman*	(*x*: 14.71, *y*: −19.71, *z*: 2.79)			2	1	1	100	30	−1.750	0.133	−0.940	−3.200	−0.800	
				3	1	1	100	29	0.200	−0.667	−0.520	3.540	−1.030	No

Continued improvement is defined as improvement in z-score at visit 3 compared to visit 2 for each cognitive test/domain. A ‘favourable’ cognitive recovery profile is defined as continued improvement in over half of the cognitive domains for each patient.

### Resting-state experiment: MEG recordings

A 157-channel axial gradiometer MEG system (Kanazawa Institute of Technology, Nonoichi, Ishikawa, Japan) was used to record magnetic fields while participants rested in a magnetically shielded room (VAC, Hanau, Germany). Recordings were collected while participants lay supine inside the MEG scanner and fixated on a cross projected onto a screen in front of them. As part of our larger protocol, one minute of eyes-open resting-state data were collected and analysed for each patient, The length of the recording was chosen to keep scan times low and is consistent with previous studies.^30–32^ Soleimani *et al*.^21^ have shown that recordings of 40 seconds or greater, with the same source space and parameter dimensions as in this work, are sufficient to identify reliable and consistent Granger Causal (GC) estimates. Resting-state MEG data were collected from the same participants again ∼6 and 12 months later (visits 2 and 3) when they returned for follow-up clinical evaluation. A sampling rate of 1 kHz was used with a 200 Hz low pass filter and a 60 Hz notch filter to remove line noise. The location of the head inside the MEG system was measured using five marker coils and the head shape was digitized using the Polhemus 3SPACE FASTRAK system. The digitized head shape and coil locations were used to obtain the mapping between the sensors onto the sources.

### Pre-processing and data cleaning

All pre-processing steps were performed using MNE-python 0.21.0.^33,34^ After excluding any noisy channels, temporal signal space separation was employed to remove artefacts.^35^ The data were then filtered between 0.1 and 100 Hz, via a zero-phase finite impulse response filter (using the default setting of MNE-python 0.21.0), after which independent component analysis^36^ was applied to remove nuisance components due to eye-blinks, facial muscle movements and cardiac artefacts. The initial 5 seconds of the data were discarded, and the subsequent 55 seconds of the data were extracted and downsampled to 50 Hz for all remaining analysis. Finally, the data were filtered within the beta band (from 13 Hz to ∼25 Hz) based on previous results showing significant differences in the beta band between stroke patients and controls.^20^ We used finite impulse response filters designed to exhibit negligible leakage to make sure that border effects in the frequency domain were minimized and chose a downsampling frequency of 50 Hz to both include the upper end of the beta band (just under 25 Hz) and reduce the runtime of the algorithm.

### Connectivity analysis

To investigate the changes in cortical connectivity across visits, we utilized the NLGC framework which identifies directed interactions between different cortical regions referred to as GC links.^21^ In brief, we say that brain region A has a directed GC link to brain region B if statistical predictions of the time-course of the activity of region B are significantly improved by using the previous activity of region A as a regressor, as compared to omitting region A from the set of regressors.^37^ According to this definition, two regions might possibly show connectivity in either direction, bidirectionally, or not at all.

The NLGC framework additionally allows the *direct inference* at the cortical source level of such GC links, from MEG data, without the need for an intermediate step of source localization, thereby significantly reducing the false detection rate incurred by older two-stage methods. In two-stage connectivity analysis methods, the source activities are first estimated via source localization, followed by identifying GC links from the estimated sources. However, statistical biases incurred during the source localization stage, primarily in the spatial extent of the estimated sources, propagate to the second stage of parameter estimation required for GC identification. This typically amplifies those biases; for instance, spatial spread in a source localization estimate may well be acceptable for that purpose, but when then used for connectivity analysis, it propagates any spatial spread error via both ends of the connectivity measure. Instead, NLGC models the underlying neural source activity via a single second-order sparse vector auto-regressive model that is mapped to the sensors via a forward model. The model parameters are then directly estimated by combining the forward model and auto-regressive estimation into a unified framework, from which the GC links are identified. NLGC automatically assesses the significance of the GC connections using the Benjamimini–Yekutieli (BY) procedure to control the false discovery rate at 0.1%. The resulting connectivity map represents significant directional GC links among 84 cortical sources (ico-1 source space), a subset of which contribute to each specific region of interest (ROI). To achieve an acceptable accuracy in the forward model while ensuring manageable runtime of the algorithm, each of the 84 cortical sources is represented by the first four principal components of corresponding neural sources located in the ico-4 source space. As python implementation of NLGC is publicly available on Github.^38^

**Figure 2:**
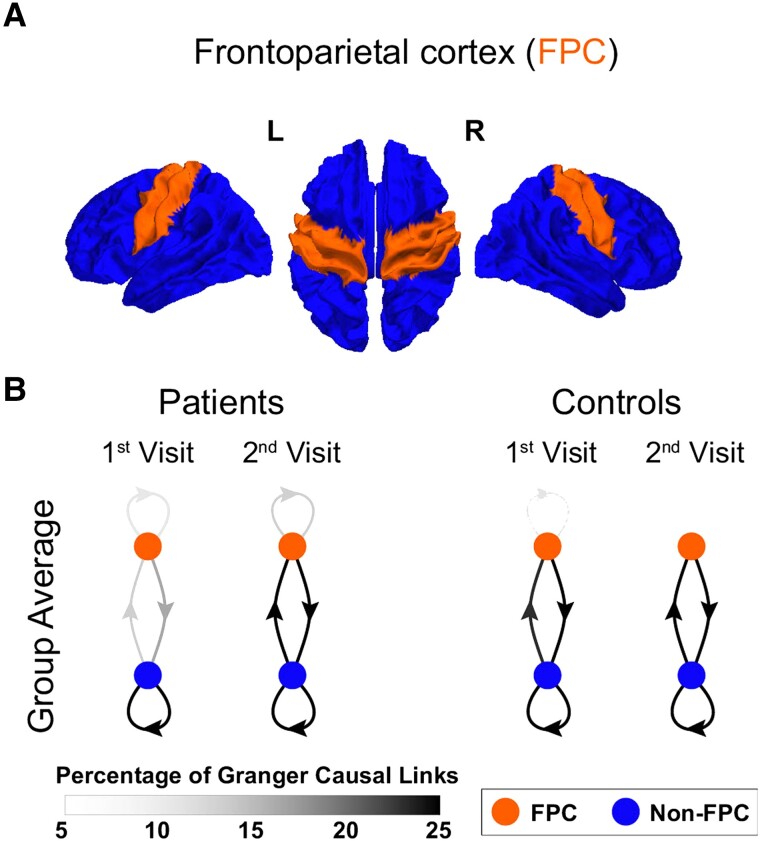
**FPC connectivity across visits.** (**A**) FPC ROI. (**B**) Directional connectivity plots between bilateral FPC (orange) and non-FPC (blue) areas. The percentage of causal links does not change significantly across visits for controls (*N* = 4). However, after stroke (*N* = 6), FPC becomes more involved in the overall connectivity by the second visit, with increased connectivity to and from non-FPC areas. The greyscale depicts the percentage of links between areas.

NLGC analysis was applied to MEG recordings from each individual for each visit. The connectivity maps were then summarized in terms of the percentage of significant GC links to/from bilateral frontoparietal cortices (FPC) including motor and premotor cortex. The FPC ROI consisted of the ‘precentral’, ‘paracentral’ and ‘postcentral’ ROIs of the Desikan–Killiany atlas.^39^ (See Fig. 2) This region was chosen for primary analysis given the bilateral impaired processing speed noted on prior clinical testing in our patient population, as well as the clinical dysexecutive syndrome observed in patients,^17^ potentially localizing to the frontoparietal network including the premotor cortex, critical for planning and executing tasks. In this study, we refer to all other ROIs as non-FPC. Our group and others have shown abnormal bilateral beta-band activity in FPC during both motor and cognitive tasks;^17,20^ therefore, we focused on the beta band for this analysis. Other frequency bands were not formally evaluated given the small sample size. As a result, for each subject at a given visit, the connectivity map for the initial analysis was explained by an array with four entries such that each entry was the percentage of GC interactions for the four connectivity types (all bilateral): FPC → FPC, FPC → non-FPC, non-FPC → FPC and non-FPC → non-FPC. The percentage of total significant links was used, rather than the absolute number of significant links, due to its statistical robustness (the absolute number of significant links can depend on the neural signal quality and noise level at the time of recording). Analysis distinguishing between ipsilesional and contralesional hemispheric connectivity was not performed at this stage, since controls were also included in this analysis.

To investigate the role of lesion lateralization in network dysfunction and the importance of directional connectivity to longitudinal recovery, our second analysis distinguished between significant GC links identified within and between the contralesional and ipsilesional hemispheres in patients. We represented the connectivity pattern of patients at each visit with a 16-entry array where each entry was the percentage of significant GC links corresponding to *Source*_1_(*hemi*_1_) → *Source*_2_(*hemi*_2_) with *Source* being either FPC or non-FPC, and *hemi* either the ipsilesional or contralesional hemisphere. Connectivity maps were compared across visits, in order to determine the patterns of change over time associated with neural recovery. Patients whose recovery was categorized as ‘favourable’ were compared to ‘unfavourable’. Given the limited dataset, longitudinal results were reported individually for each patient.

### Statistical analysis

Paired sample t-tests with Bonferroni correction^40^ were also reported, comparing: (1) differences in the distribution of significant links between patients and controls at each visit, (2) differences across visits for each group and (3) differences between those with a favourable versus unfavourable long-term outcome.

## Results

### Analysis 1

#### Overall connectivity differences between stroke patients and controls

The connectivity patterns for each group (stroke patients and controls) were consistent across individuals and, in line with previous studies of a mild stroke, independent of lesion location.^17,20^ At visit 1, there was a significant reduction in connections between the bilateral FPC and non-FPC regions compared to controls, regardless of connectivity direction, as well as significant differences in connectivity between non-FPC areas (Table 2). Plots of extracted GC connectivity networks, showed a significantly lower proportion of connections both incoming and outgoing from FPC at visit 1 for patients compared to controls (Fig. 2). The GC network pattern remained stable across visits for the control population; connectivity changes at both the group and individual level were not significant. However, the involvement of bilateral FPC in the overall cortical network significantly increased by the second visit for stroke patients, appearing more similar to the control group (see Fig. 2 and Table 2 for full details regarding differences in connectivity between groups and changes over time).

**Table 2:** Connectivity profiles

	Patients (*P* values)		Controls (*P* values)		Patients v Controls (*P* values)			Favourable v unfavourable (*P* values)		
Connectivity	First versus second	Second versus third	First versus second	Second versus third	Visit 1	2	3	Visit 1	2	3
Non-FPC –> FPC	*** (0.00047)	NS (0.06)	NS (0.43)	NS (0.41)	* (0.04)	NS (0.08)	NS (0.71)	NS (0.55)	NS (0.48)	** (0.008)
FPC –> Non-FPC	*** (0.00072)	NS (0.11)	NS (0.35)	NS (0.39)	* (0.03)	NS (0.14)	NS (0.29)	NS (0.5)	NS (0.78)	* (0.04)
FPC –> FPC	* (0.043)	NS (0.26)	NS (0.15)	NS (0.18)	NS (0.8)	* (0.024)	NS (0.4)	* (0.03)	NS (0.46)	NS (0.92)
Non-FPC –> Non-FPC	*** (0.0001)	* (0.02)	NS (0.36)	NS (0.4)	** (0.004)	** (0.008)	NS (0.81)	NS (0.31)	NS (0.96)	** (0.0013)

	Favourable (*P* values)	Unfavourable (*P* values)
Non-FPC –> FPC	* (0.012)	NS (0.09)
FPC –> Non-FPC	** (0.004)	NS (0.44)
FPC –> FPC	NS (0.31)	NS (0.39)
Non-FPC –> Non-FPC	*** (0.0007)	NS (0.11)

The table shows significant differences (*P* values) in connectivity. The bump out highlights differences between stroke patients with favourable versus unfavourable recovery profiles at visit 3. NS, not significant.

*P < 0.05.

**P < 0.01.

***P < 0.001.

#### Favourable versus unfavourable long-term recovery

**Figure 3:**
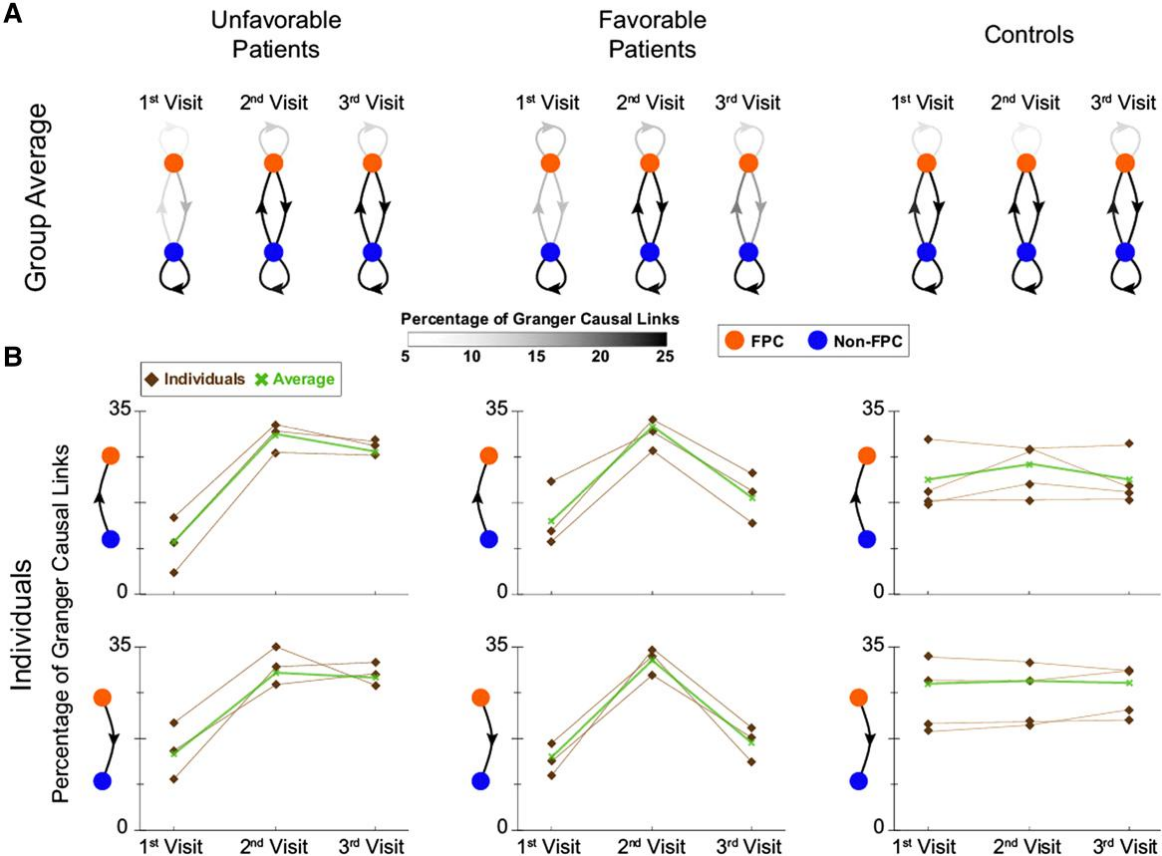
**Longer-term FPC connectivity changes.** (**A**) Directional functional connectivity plots illustrate significant differences between patients with a favourable (*N* = 3) versus unfavourable (*N* = 3) long-term recovery profile with respect to the FPC’s = orange role in functional connectivity between visits 2 and 3. Controls (*N* = 4) remain constant. (**B**) Graphs of the percentage of links at visits 1–3 for individual controls and patients mirror group results and illustrate that all stroke patients show an initial uniform increase in FPC’s involvement by visit 2, followed by a pronounced decrease for those with continued recovery compared to other groups.

While stroke patients exhibited consistent overall clinical improvement between visits 1 and 2, along with increased connectivity involvement of FPC, subsequent clinical recovery observed at visit 3 was variable (Table 1). Three patients continued to show multi-domain improvement—a long-term ‘favourable’ recovery profile; while two others performed worse overall than at visit 2—defined as ‘unfavourable’. Improvement on the expanded cognitive battery mirrored changes in MoCA score, so patient 4, who returned for the MEG neural scan but not full clinical testing, and was noted to have a deterioration of performance on the MoCA between visits 2 and 3, was also classified as ‘unfavourable’ long-term. Connectivity patterns differed between these two groups. Figure 3A demonstrates how both groups show consistent initial patterns of recovery at visit 2 that appear more similar to the distribution of causal links displayed by controls that remain consistent across visits. Group averages mirrored individual results (Fig. 3B). Interestingly, at visit 3, the pattern of connectivity continued to evolve for those demonstrating additional recovery (a ‘favourable’ clinical profile). This appears less so to be the case for those with an ‘unfavourable’ clinical profile and prompted us to pursue further analysis of patients using ipsi- and contralesional regions to explore the changes in inter- and intrahemispheric connections.

### Analysis 2

#### Inter-hemispheric directional functional connectivity over time

**Figure 4:**
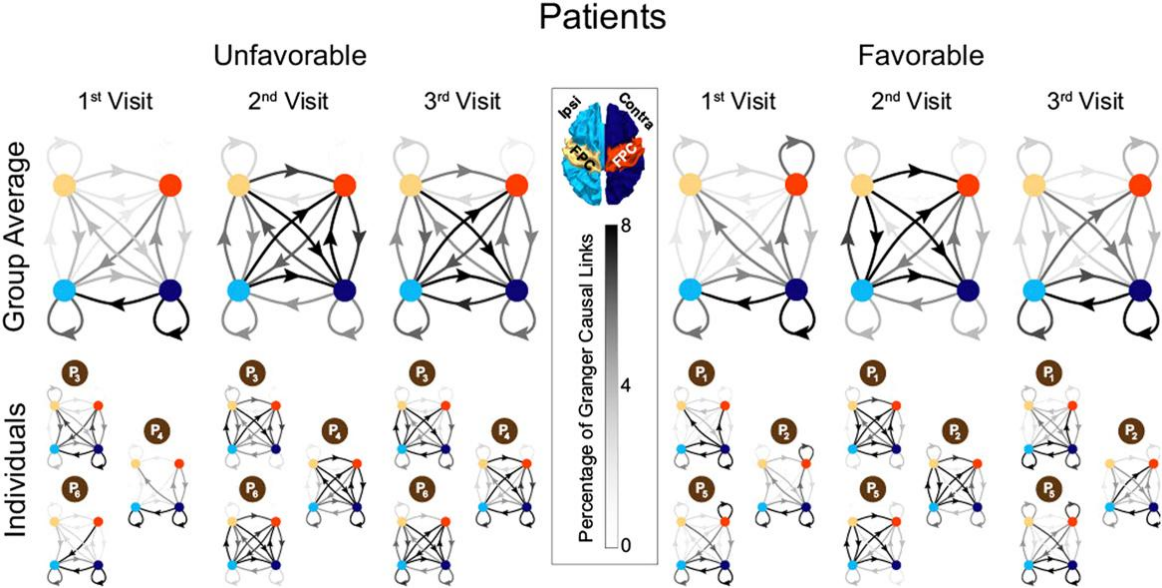
**Bilateral hemispheric connectivity changes.** Directional functional connectivity plots show individual and group results. At visit 2, patients with both a favourable (*N* = 3) and unfavourable (*N* = 3) long-term recovery profile show an increase in connections from ipsilesional FPC (yellow) to contralesional FPC (orange) and contralesional non-FPC (dark blue) areas that parallels a uniform clinical improvement. In addition, there is increased connectivity between ipsilesional non-FPC (light blue) and contralesional non-FPC (dark blue). This pattern remains similar for patients with an unfavourable recovery at visit 3; however, for those with a favourable outcome, there is a relative decrease in connectivity from ipsilesional FPC (yellow) to the contralesional hemisphere in favour of increased connectivity from contralesional non-FPC (dark blue) to ipsilesional non-FPC (light blue). The proportion of connections is represented by the grey scale.

Only stroke patients were analysed at this level (necessary for defining ipsilesional and contralesional hemispheres). Individual connectivity maps and group averages are displayed in Fig. 4. Within-patient group averages appear an accurate reflection of each group as a whole, with the similarities across subjects in the group being more apparent than the differences (this is critical due to the small sample size of the cohort). For both groups, there was an increase in the percentage of links from ipsilesional FPC to contralesional areas (both FPC and non-FPC). Importantly, those who continued to improve with favourable cognitive profiles at visit 3 demonstrated a further shift in directional functional connectivity with statistically significant differences in links from multiple areas over time not seen by the unfavourable group. (Table 3) Specifically:

- FPC → FPC was weak in both directions at visit 1. At visit 2, Ipsilesional → Contralesional connections were enhanced for all patients. By visit 3, Ipsilesional → Contralesional was diminished more for favourable patients than unfavourable.
- Ipsilesional non-FPC → Contralesional FPC was also weak at visit 1 for all patients. While it became stronger and bidirectional at visit 2, for favourable patients this relationship continued to evolve and was unidirectional (strongly Ipsilesional → Contralesional) by visit 3.
- Ipsilesional FPC → Contralesional non-FPC was weakly bidirectional at the 1st visit for both groups but at the second visit, the groups diverged (unfavourable patients displayed strongly bidirectional connectivity while favourable displayed unidirectional Ipsilesional → Contralesional). By visit 3, in unfavourable patients Contralesional → Ipsilesional weakened but did not disappear, whereas it remained almost absent for the favourable group.
- Non-FPC → Non-FPC was strongly Contralesional → Ipsilesional at the first visit, reversed at the second visit, and became strongly bidirectional at the third visit for both groups.

**Table 3:** Laterality and inter-hemispheric connectivity

		Second versus third visit (*P* values)		Favourable versus unfavourable (*P* values)		
Connectivity		Favourable	Unfavourable	First	Second	Third
Non-FPC -> FPC	Ipsi - Ipsi	NS (0.11)	NS (0.4)	NS (0.18)	NS (0.39)	NS (0.1)
	Contra—Contra	NS (0.32)	NS (0.2)	NS (0.39)	NS (0.26)	NS (0.38)
	Ipsi—Contra	* (0.047)	NS (0.5)	NS (0.058)	NS (0.33)	NS (0.052)
	Contra—Ipsi	NS (0.38)	NS (0.1)	NS (0.38)	* (0.017)	NS (0.65)
FPC -> Non-FPC	Ipsi—Ipsi	** (0.008)	NS (0.3)	NS (0.18)	* (0.02)	NS (0.06)
	Contra—Contra	NS (0.5)	NS (0.051)	NS (0.051)	*** (0.0003)	* (0.012)
	Ipsi—Contra	** 0(0.009)	NS (0.41)	NS (0.072)	NS (0.34)	** (0.004)
	Contra—Ipsi	NS (0.14)	NS (0.42)	NS (0.18)	NS (0.36)	NS (0.28)
FPC -> FPC	Ipsi—Ipsi	NS (0.33)	NS (0.3)	NS (0.058)	NS (0.5)	NS (0.39)
	Contra—Contra	NS (0.07)	NS (0.058)	* (0.042)	NS (0.18)	NS (0.062)
	Ipsi—Contra	** (0.002)	NS (0.12)	NS (0.11)	NS (0.32)	NS (0.08)
	Contra—Ipsi	NS (0.17)	NS (0.21)	NS (0.38)	NS (0.25)	NS (0.5)
Non-FPC -> Non-FPC	Ipsi—Ipsi	NS (0.06)	NS (0.07)	NS (0.5)	NS (0.38)	NS (0.38)
	Contra—Contra	NS (0.06)	NS (0.14)	NS (0.27)	NS (0.31)	NS (0.15)
	Ipsi—Contra	NS (0.19)	NS (0.36)	NS (0.065)	NS (0.39)	NS (0.5)
	Contra—Ipsi	*** (0.0001)	NS (0.06)	NS (0.07)	NS (0.4)	*** (0.0001)

The table shows significant differences (*P* values) in connectivity between the ipsilesional and contralesional hemisphere in stroke patients and illustrates the differences between those with favourable and unfavourable long-term outcomes, particularly at visit 3. NS, not significant.

* P < 0.05, ** P < 0.01, *** P < 0.001.

Also of note, connections between the contralesional FPC and non-FPC were weakly directional (in favour of non-FPC → FPC) at the 1st visit for both groups, but strongly bidirectional for unfavourable patients over time while remaining weakly directional (non-FPC → FPC) for favourable. Overall, the variable evolution of directional functional connectivity across groups over time resulted in numerous significant differences between groups at visit 3. The continued evolution of the favourable group resulted in fewer inter-hemispheric connections, while the unfavourable group continued to rely heavily on these links.

## Discussion

This proof-of-concept study using resting-state MEG to evaluate the relationship between neural connectivity and cognitive dysfunction in individuals, both acutely and longitudinally after a minor stroke, supports the hypothesis that impaired functional connectivity is associated with clinical symptoms. Following a stroke, an abnormal pattern is consistent across patients, independent of lesion location, as suggested indirectly by our previous work.^17,20^ Our data show that there is a significant decrease in the percentage of connections going into and away from FPC bilaterally acutely in stroke patients compared to controls. This lack of involvement of these key brain regions, important for planning and executing responses during tasks, may explain delayed reaction times. The increased involvement of FPC bilaterally seen at visit 2, corresponding to clinical improvement, suggests this may be the case. Furthermore, our data suggest the importance of laterality and continued changes in directional inter-hemispheric functional connectivity for the longitudinal recovery of cognitive networks and improvement of symptoms over time. We have shown that improvement in performance at both 6- (visit 2) and 12 months (visit 3) post-stroke is associated with the persistent evolution of intra- and inter-hemispheric connections: with initially increased reliance on connections towards the contralesional hemisphere that becomes less over time for individuals with a favourable long-term recovery profile.

These specific results are consistent with broader results from previous studies investigating motor recovery. Analysis of resting-state functional connectivity evaluated using M/ electroencephalography (EEG) has shown that control populations exhibit a more balanced network compared to disrupted frontoparietal connectivity observed in stroke patients with motor deficits,^41,42^ predominantly with respect to inter-hemispheric coupling.^43,44^ Improved function in those participants’ weakness had a direct relationship with strength and the number of inter-hemispheric connections in FPC.^45–48^ It is important to also note that the strokes within those cohorts were clearly located within motor pathways, which was not the case in our sample, and that motor performance was the primary outcome measure.

Other connectivity studies have focused on stroke patients with language deficits. Some reported abnormal neural dysfunction in perilesional areas acutely after infarct,^49,50^ but a recent review of prior fMRI studies of aphasic patients found reports of disruption of inter-hemispheric connections in auditory and language networks.^51^ Our current study substantially broadens these results by demonstrating that disruptions of connectivity are associated with cognitive dysfunction even in patients without other significant cortical deficits such as hemiparesis or aphasia. Thus, it illustrates that it is specifically the disruption of the network (altered connectivity), rather than dysfunction within specific cortical areas, that is associated with poor cognitive performance.^23,52^ Furthermore, the consistent clinical phenotype observed within our cohort, which is independent of lesion location and irrespective of infarct size indicates the importance of network integrity to perform cognitive tasks.

Our study not only illustrates that broad network dysfunction can occur with a single subcortical infarct but begins to elucidate the compensatory pathways that may be associated with clinical improvement over time. While prior work has evaluated functional connectivity alterations in acute stroke patients undergoing rehabilitative intervention, the role of functional connectivity changes in the natural recovery of cognitive deficits in patients with minor stroke is not fully understood. These mechanisms appear to be independent of lesion size or location and initially involve an increase of signals towards the contralesional hemisphere. Interestingly, it appears that in order for patients to continue to improve, it is necessary for many of these connections to decrease over time, potentially indicating more reliance on the recovering ipsilesional hemisphere at visit 3 in those with optimal recovery profiles. This concept of continued reorganization of function during the subacute period has been proposed previously with respect to language recovery^35^ and has already been described in the literature as a potential recovery mechanism based on transcranial magnetic stimulation, Wada testing and cortical neurostimulation.^53^ Our study is novel in its inclusion of subcortical infarcts and focuses on network dynamics observed using MEG to evaluate cognition. Importantly, the findings parallel those of prior connectivity studies using fMRI, which have demonstrated both inter- and intrahemispheric abnormalities during the early stages of recovery^54^ that normalize with good recovery.^52^

Other studies have focused on extracting network-level changes over time, specifically in the motor cortex of acute stroke patients, utilizing various neuroimaging techniques including fMRI, MEG, and EEG.^55–58^ The high spatial resolution of fMRI has shown a bilateral reduction of connections between the primary motor cortex in patients with chronic stroke compared to healthy individuals.^59^ After a one-month rehabilitative intervention, connectivity between ipsilesional and contralesional primary motor cortices significantly increased. While many studies have suggested that dynamic changes in the connectivity pattern of the motor cortex^60,61^ mainly involve inter-hemispheric connections,^62,63^ other studies have found a correlation between motor recovery and functional connectivity strength restoration in frontoparietal, or sensorimotor cortex, mostly in the ipsilesional hemisphere.^64–66^ In a population similar to ours, using fMRI to evaluate GC, Allegra *et al.* also found abnormalities in inter-hemispheric connections following a stroke that when improved, correlated with clinical improvement.^58^ This supports the theory that the resolution of impaired directional functional connectivity is important for recovery. Similarly, disruption of inter-hemispheric connectivity irrespective of lesion location or size has also been reported previously after stroke in patients with motor impairment using fMRI,^62^ as well as hemispatial neglect. Critically, however, using MEG allows us to evaluate cognitive processes that occur on a millisecond scale, and, furthermore, in our study infarcts did not involve any eloquent cortex or areas traditionally associated with cognitive impairment. We demonstrate that a single subcortical lesion, independent of location, is enough to disrupt generalized connectivity in a predictable way. Whether these changes are modifiable with rehabilitation paradigms, non-invasive stimulation, or pharmacotherapy, remains to be seen. Further exploring the compensatory network alterations that must occur in order to allow individuals to continue to recover is an important first step towards developing and testing effective treatment strategies.

Previous work in physiological functional connectivity analyses can be categorized based on methodology into two groups: source- and sensor-level connectivity analysis. While the former needs an intermediate source localization step to estimate the source activities followed by connectivity inference, the latter takes advantage of the putative relationship between locations of the sensors and cortical areas to interpret the connectivity patterns. However, both approaches are known to suffer from false detections and spatial mis-localizations, especially in resting-state studies.^67–69^ By using the NLGC framework, these shortcomings are addressed by directly inferring the cortical GC links from the MEG data, without resorting to intermediate localization estimates. NLGC also addresses a common shortcoming of existing directional connectivity analyses which typically require long data durations to be able to uncover the underlying connections reliably at a low false detection rate. Our results here show that the identified GC networks are consistent at both the individual and group levels, thus demonstrating that 55 seconds of high temporal resolution resting-state data MEG data suffices to reliably detect GC networks in the beta frequency band, consistent with previous validation analyses.^21^

For this population of individuals with minor stroke and cognitive impairment, we chose to evaluate functional connectivity using MEG. fMRI, with its relatively low temporal resolution (i.e. seconds not milliseconds), cannot capture critical neural processes such as beta-band activity in the motor cortex.^70^ Such beta-band activity, which is known to be critical for processing speed and the motor planning needed to generate responses,^71,72^ is easily captured with M/EEG.^73,74^ Unusual beta-band activity has been detected in patients with motor deficits, including those suffering from stroke;^75^ however, Kulasingham and colleagues^20^ recently also observed that patients with only minor stroke and abnormal processing speed but no significant hemiparesis demonstrated similar changes. The reduced bilateral Rolandic beta activity during the recovery period irrespective of lesion location (and most notably with strokes outside of the motor pathway), suggests that even such small and distant lesions may result in global network impairment, which is consistent with our findings. It is important to note that those abnormalities in beta power persisted regardless of clinical improvement, illustrating that power within the beta band alone is not driving behaviour, or responsible for the continued evolution of directional functional connectivity observed in this study. Interestingly, measures of connectivity in the beta band have also been implicated in contributions to fMRI measures of connectivity,^76^ but here they can be seen directly.

Critically, our results demonstrate consistent connectivity patterns in the control group across visits, as expected. In contrast, the detected GC networks for the patients’ first visit exhibit significantly reduced FPC involvement in cortical connectivity. By the second visit, the GC connections involving FPC markedly resemble those of the control group, with enhanced connections bidirectionally, but particularly towards the contralesional hemisphere. This may indicate the flow of information away from the damaged hemisphere as a potential mechanism for compensation within the network. By visit three, there are fewer of these connections and less reliance on communication with the contralesional hemisphere, perhaps as the damaged hemisphere begins to heal and increases its role in network dynamics once again for those who continue to improve, demonstrating the need for the continued evolution of the network to optimize recovery. Importantly, there appeared to be divergent patterns of directional functional connectivity between those with favourable versus unfavourable long-term recovery profiles, even early on, though most pronounced at visit 3. These network-level functional changes are strong candidates for the compensatory mechanisms of cognitive recovery, and their presence on MEG suggests it may be a useful biomarker of recovery or even a potential predictor of longer-term function and is useful clinically. It also suggests that augmentation of the network through pharmacologic mechanisms or neurostimulation, enhancing or inhibiting input from specific areas at various stages of recovery, may help to hasten or augment improvement.

This study does have limitations. It is a small sample size, consisting of six patients and four controls from a single center. Strokes are heterogeneous in their location. However, the pattern of clinical deficits, as well as the pattern of neural activity measured by MEG, is consistent across patients, independent of stroke location, and distinct from the control group. The NLGC methodology demonstrates consistent robust results even in such a small sample, that is consistent with prior studies evaluating similar brain regions, as well as prior indirect evidence in patients with minor stroke symptoms. In addition, patients were divided into ‘favourable’ and ‘unfavourable’ long-term recovery based on their overall multi-domain performance but had some variability. While we were able to directly illustrate the overall consistency between groups by adding longitudinal plots of the directional connectivity, a larger study would be needed to evaluate the connectivity of specific cognitive networks that may affect individual task performance. Finally, while network-level functional changes may represent compensatory mechanisms and serve as a potential target for future augmenting therapies, we lack the ability to conclusively determine their significance and whether they represent the cause or effect of recovery patterns.

Despite these limitations, our findings support many key concepts: (1) early post-stroke cognitive dysfunction appears to be associated with impaired functional connectivity that is independent of lesion location in individuals with minor stroke; (2) an increase in inter-hemispheric connections, with initial reliance on an increase in the connections between hemispheres, is associated with clinical improvement months after recovery; (3) MEG may be a useful biomarker to explore connectivity changes associated with the recovery and surrogate outcome metric for future treatment trials, though further studies with a larger sample size are needed to determine if the number of inter-hemispheric links is directly correlated to the degree of clinical improvement and whether it may also have a role in *predicting* long-term outcome. A larger study, evaluating additional frequency bands and specific cognitive networks is needed, along with secondary analyses evaluating the implications of stroke laterality on recovery patterns.

## Data Availability

Full MEG and clinical data are available from the corresponding author upon request.

## Abbreviations

BY =: Benjamimini–Yekutieli
EEG =: electroencephalography
fMRI =: functional magnetic resonance imaging
FPC =: frontoparietal cortex
GC =: granger causality
MEG =: magnetoencephalography
MoCA =: Montreal cognitive assessment
mRS =: modified Rankin scale
NIHSS =: National Institutes of Health Stroke Scale
NLGC =: network localized granger causality
ROI =: region of interest
